# Excited state dynamics of bis-dehydroxycurcumin *tert*-butyl ester, a diketo-shifted derivative of the photosensitizer curcumin

**DOI:** 10.1371/journal.pone.0175225

**Published:** 2017-04-27

**Authors:** Luca Nardo, Angelo Maspero, Andrea Penoni, Giovanni Palmisano, Erika Ferrari, Monica Saladini

**Affiliations:** 1 Department of Medicine and Surgery, University of Milano Bicocca, Monza, Monza and Brianza, Italy; 2 Department of Science and High Technology, University of Insubria, Como, Como, Italy; 3 Department of Chemical and Geological Sciences, University of Modena and Reggio Emilia, Modena, Modena, Italy; University of Lincoln, UNITED KINGDOM

## Abstract

Bis-dehydroxycurcumin *tert*-butyl ester (K2T23) is a derivative of the natural spice curcumin. Curcumin is widely studied for its multiple therapeutic properties, including photosensitized cytotoxicity. However, the full exploitation of curcumin phototoxic potential is hindered by the extreme instability of its excited state, caused by very efficient non radiative decay by means of transfer of the enolic proton to the nearby keto oxygen. K2T23 is designed to exhibit a tautomeric equilibrium shifted toward the diketo conformers with respect to natural curcumin. This property should endow K2T23 with superior excited-state stability when excited in the UVB band, i.e., in correspondence of the diketo conformers absorption peaks, making this compound an interesting candidate for topical photodynamic therapy of, e.g., skin tumors or oral infections. In this work, the tautomeric equilibrium of K2T23 between the keto-enolic and diketo conformers is assessed in the ground state in several organic solvents by UV-visible absorption and by nuclear magnetic resonance. The same tautomeric equilibrium is also probed in the excited-state in the same environments by means of steady-state fluorescence and time-correlated single-photon counting measurements. These techniques are also exploited to elucidate the excited state dynamics and excited-state deactivation pathways of K2T23, which are compared to those determined for several other curcuminoids characterized in previous works of ours. The ability of K2T23 in photosensitizing the production of singlet oxygen is compared with that of curcumin.

## Introduction

**Fig 1:**
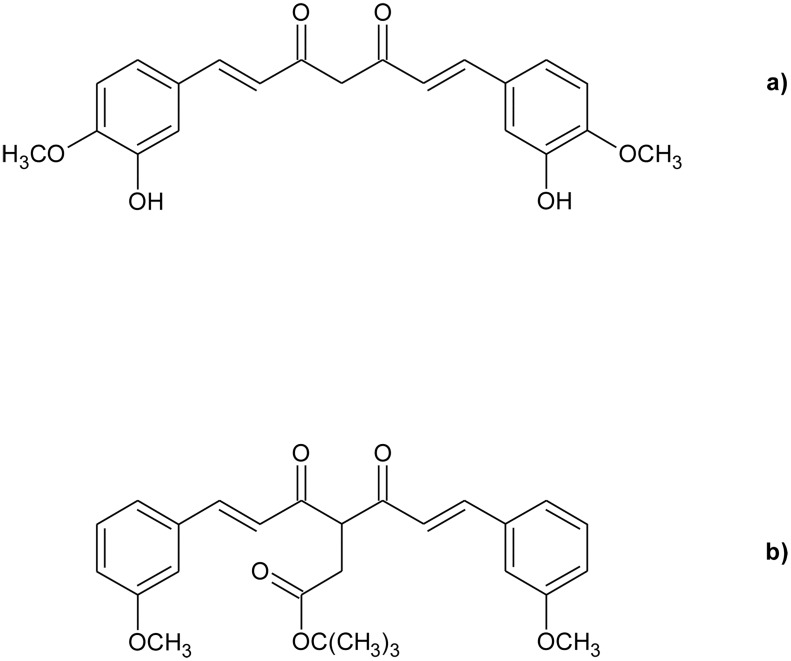
Curcuminoids structures. Molecular structures of a) curcumin and b) K2T23.

Curcumin (CURC, Fig 1a) has been recently demonstrated to be endowed with notable phototoxic potency on both bacterial and human cancer cell lines [1–12]. This property, combined with its edibility at doses of several grams per day and high tolerance at the systemic level [13], makes CURC and its derivatives a particularly promising class of model compounds for the development of photo-activated drugs, although the notable instability of the CURC excited state constitutes a drawback in view of its formulation into a photosensitizer.

Curcuminoids, alike other β-diketones, exhibit tautomerism between several keto-enolic and diketo structures [14]. CURC prevalently assumes the H-bonded closed *cis* enol conformation both in the gas phase and in non-polar solvents due to formation of an intramolecular H-bond between the keto oxygen and the enol proton (keto-enol H-bond, KEHB) [15–19]. Moreover, although in polar solvents the KEHB is efficiently perturbed by solute-solvent interactions, the open *cis* and *trans* enol structures prevail, respectively, in weakly- and strongly-H-bonding solvents [17,19]. Traces of *trans* (*anti)* diketo conformer, which is as much as 10-fold less polar than the enol forms, are detectable in non-polar solvents [15,19], while the CURC *cis* diketo conformer, although being endowed with a molecular dipole moment 10 times higher than those of the enols, is unstable even in polar solvent [15,18]. In conclusion, at room temperature, the tautomeric equilibrium of solubilized CURC is totally shifted towards enol conformers independent from the solvent properties. This tautomeric equilibrium is shared also by several other curcuminoids modified at the phenyl rings, which we have investigated in previous studies [20–24].

In the above-quoted works, we introduced a model to interpret the fluorescence decays measured for CURC and to foresee the effects of substitutions on the excited-state dynamics of symmetrically phenyl-modified CURC analogues. In particular we demonstrated that a particularly fast excited-state intramolecular proton transfer (ESIPT) mechanism takes place between the enol and keto groups of the β-diketone moiety when curcuminoids are in the closed *cis* enol conformation, i.e. in non-polar solvents. ESIPT occurs in hundreds of picoseconds for all the phenyl-substituted compounds we characterized even in polar environment, although its rate is moderated by the need of previous rearrangement of solvent molecules in the excited state in order for the curcuminoid to recover the H-bonded closed *cis* enol conformation. The solvent rearrangement moderated ESIP rate is still much faster than radiative decay rate, thus constituting a potent channel of excited-state deactivation competing with photosensitization.

In the recent past we tried to overcome this issue by substituting the β-diketo system with a cyclohexanone ring, obtaining a compound, cyclovalone, whose excited state dynamics were actually slower than those typical of β-diketo curcuminoids, which was reflected by enhanced production of reactive oxygen species (ROS) upon weak photoexcitation. However, cyclovalone was extremely photolabile, thus CURC resulted a more performing photosensitizer even at moderate light doses [25].

Another strategy was suggested by some works of Gilli et al. [26,27]. These authors observed that, for several simple β-diketones, the tendency to assume the keto-enolic structures and form the KEHB was notably inhibited by the presence of carbonyl substituents. Obviously, *S*_1_ deactivation by means of ESIPT cannot take place in any of the diketo structures. Furthermore, for a given curcuminoid the *cis* diketo conformer should be relatively hydrophilic with respect to enol conformers, due to its superior polarity, H-bonding ability and resistance to hydrolytic dissociation [15,28]. This might also be of interest as one major challenge toward full exploitation of CURC medicinal properties lays in the poor water solubility of the compound at acidic pH and its fast hydrolysis at neutral and basic pH [29,30]. Accordingly, we have previously synthesized K2A23, a curcuminoid bearing a carboxylic acid substituent at the carbonyl [28] and tested its excited-state dynamics [31]. Indeed, K2A23 displayed a keto-enol equilibrium notably shifted towards the diketo structures with respect to CURC both in the ground and in the excited state. Consequently, the compound warranted enhanced water solubility. Moreover, K2A23 lacked the hydroxyl phenolic substituents which are involved in the inter-molecular charge transfer mechanisms leading to the fast decay of CURC in H-bonding environment [22,24]. However, although the diketo conformers exhibited slower excited state deactivation dynamics with respect to the enol tautomers, in the case of K2A23 the stabilizing effect on the *S*_1_ excited-state caused by the shift of the keto-enolic equilibrium was more than counterbalanced by a destabilizing one, induced by an unexpected decay mechanism involving a displacement of the carboxyl proton with respect to the carboxyl oxygen [31].

In this work, we consider another carbonyl-modified curcuminoid, K2T23, in which the carboxylic acid moiety of K2A23 is substituted by a rather inert *tert*-butyl ester group, incapable of forming H-bonding patterns with solvent molecules which in turn might lead to undesired excited-state deactivation pathways [28]. The equilibrium between the enol and diketo conformers of K2T23 is determined in a range of organic solvents of varying polarity and H-bonding properties by means of UV-visible absorption spectroscopy and nuclear magnetic resonance (NMR). The stability of the K2T23 *S*_1_-state is evaluated by means of steady-state fluorescence and time-correlated single-photon counting (TCSPC) experiments. The excited-state behavior is explained by attributing an excited-state deactivation pathway to each of the exponential transients detected in the fluorescence decay distributions. Finally, the ability of K2T23 in photosensitizing the production of ROS upon excitation at either the diketo or the keto-enol conformers absorption peaks is compared to that of CURC in methanol, ethanol, dimethylformamide and acetonitrile.

## Materials and methods

### Chemicals and sample preparation

K2T23 was synthesized according to the procedure described in [28]. The solvents used for the absorption and fluorescence experiments are listed in Table 1. They were ≥ 99.5% pure and were used as received.

**Table 1:** Chemical-physical properties of the used solvents.

Environment	Solvent	ε	α	β
Non-polar	Cyclohexane	2.02	0	0
Polar weakly-H-bonding	Chloroform	4.81	0.44	0
	Dichloromethane	8.93	0.13	0.10
	Acetonitrile	38.8	0.19	0.31
H-bond acceptors	Dimethylformamide	37.6	0	0.69
	Dimethylsulfoxide	48.9	0	0.76
Alcohols	Isopropanol	19.92	0.78	0.95
	Ethanol	25.07	0.83	0.77
	Methanol	33.62	0.93	0.62

In Table 1 we also report the values of dielectric constant, ε, and Kamlett-Tafft acidity parameter, α, and basicity parameter, β[32]. The NMR spectra were measured in deuterated cyclohexane, chloroform, dichloromethane, acetonitrile, dimethyl sulfoxide, and methanol.

### Steady-state spectroscopy

The UV-VIS absorption spectra were recorded with a Perkin Elmer Lambda 2 UV-VIS spectrophotometer. The fluorescence emission and excitation spectra were measured with a PTI Fluorescence Master System spectrofluorimeter interfaced with a proprietary acquisition software performing real-time correction with respect to the excitation lamp and the detector spectral responses. Fluorescence quantum yields were estimated by comparison with a solution of dimethyl-popop in ethanol excited at 363 nm (Φ_Fl_ = 0.95, [33]), upon suitable normalization with respect to both the relative absorption of the specimens and the refractive index of the solvents. The quantum yield values reported in the Results and Discussion section (*vide infra*) are the average over three parallel samples, with errors given by the pertaining standard deviation. It is worth noting that such errors probably underestimate the actual errors on the absolute quantum yield values. However, because throughout the manuscript we only compare the quantum yields recorded with the same method and instrumentation in different solvents and upon excitation at different wavelengths, we deem it primarily relevant to provide the reader with information about the reproducibility of the values, rather than on their preciseness.

NMR measurements were performed with a Bruker ADVANCE 400 spectrometer. ^1^H spectra were acquired at 400 MHz at room temperature (T = 298 K), with pulse delay 10 s, on 1.5 10^−2^ *M* solutions of K2T23 in the deuterated solvents quoted above.

### Time-resolved fluorescence

The fluorescence decay patterns were reconstructed by means of a TCSPC apparatus endowed with <30 ps temporal resolution, which is described in more details in [34]. The fluorescence of the solutions was excited in proximity of the keto-enol conformers absorption peak (*vide infra*) by means of the built-in second harmonic (420 nm wavelength) of a SESAM mode locked Ti:Sapphire laser (Tiger ps SHG, Time Bandwidth Products, Zurich, CH), and in proximity of the diketo conformers absorption peak (*vide infra*) by generating out of cavity the Ti:sapphire third harmonic (280 nm) as described elsewhere [31]. The rough fluorescence decay distributions were fitted to a multi-exponential decay function added with a constant background, exploiting the Levenberg-Marquardt *χ*^2^ minimization algorithm provided by the data analysis software Origin 7. For each decay pattern, the number of decay components was established by adding components one by one until further addition resulted in determination of more than one component with the same decay constant. The decay times and relative amplitudes reported in the Results and Discussion section are the averages of the values obtained from the fits of three measured decay patterns; the associated errors are the corresponding standard deviations.

### Spectrofluorimetric detection of photosensitized ROS generation

The production of ROS by K2T23 was compared to that of CURC in methanol, ethanol, acetonitrile and dimethylformamide by using the fluorescent indicator 9,10-dimethylanthracene (Fluka). Solutions at 0.5 μM photosensitizer (*i*.*e*., CURC or K2T23) concentration and 5 μM concentration of 9,10-dimethylanthracene were prepared immediately before the experiments. The fluorescence emission of 9,10-dimethylanthracene was measured by means of the PTI fluorimeter upon excitation at 360 nm in the emission band 380–550 nm, with the lamp intensity set at 70 W and the excitation and emission slits set at 1 nm band-pass. Once measured the fluorescence spectra of a freshly prepared solution, the same instrument was used to induce photosensitization of ROS through illumination at either 280 nm or 420 nm. To this aim, the sample was exposed for 10 min to the light produced by the PTI spectrofluorimeter lamp set at 85 W power, with excitation slits set at 20 nm band-width peaked at the desired wavelength. Blank solutions without indicator were also prepared in order to subtract the fluorescence signal produced in the indicator emission band due to out of peak excitation of the photosensitizers. The intrinsic tendency of the compounds to photodegradation was also assessed by submitting solutions of pure CURC, K2T23, or 9,10-dimethylanthracene in all the solvents used for ROS generation measurements to the same light dose as the photosensitizer/indicator mixtures. The compounds did not appreciably change their fluorescence emission properties after exposure, indicating that no severe photodegradation took place.

## Results and discussion

### Ground-state equilibrium

#### UV-Vis absorption spectroscopy

**Fig 2:**
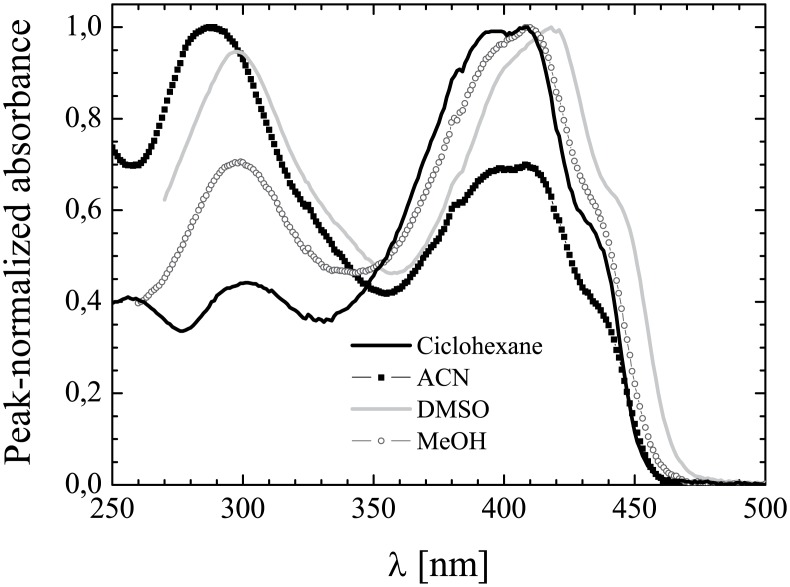
UV-Vis absorption of K2T23. Absorption spectral line-shapes of K2T23 dissolved in cyclohexane (line), acetonitrile (squares), dimethylsulfoxide (crosses), and methanol (circles).

The UV-Vis absorption spectra of K2T23 were characterized by two main transition bands, peaked at λ_DK_ ≈ 290 nm and λ_KE_ ≈ 410 nm, respectively, in all the solvents of Table 1. Exemplary spectra are plotted in Fig 2.

The peak absorption wavelengths are reported in Table 2. Led by the same considerations discussed at length in the work on K2A23 [31], we ascribe the band peaked at λ_DK_ to the presence, in all the solvents, of a sizeable fraction of K2T23 diketo conformers. This finding suggests that the *tert*-butyl ester functionalizing the carbonyl efficiently perturbs the resonance of the double bonds in the keto-enol system, thus destabilizing the keto-enol structures. As shown in [31], the relative absorbance of the diketo conformers, *A*_DK_, is a good estimation of the fraction *f*_DK_ of compound in either of the diketo conformers as far as tautomerization does not dramatically affect the molar extinction coefficient.

**Table 2:** Absorption properties of K2T23.

Solvent	*λ*_DK_ [nm] (*A*_DK_)	*λ*_KE_ [nm]
Cyclohexane	255; 301 (0.46)	401
Chloroform	275 (0.38)	411
Dichloromethane	301 (0.36)	412
Acetonitrile	290 (0.59)	411
Dimethylformamide	286 (0.57)	415
Dimethylsulfoxide	298 (0.49)	418
Isopropanol	304 (0.24)	411
Ethanol	296 (0.47)	411
Methanol	299 (0.41)	409

Peak absorption wavelengths of the diketo (*λ*_DK_) and enol (*λ*_KE_) conformers of K2T23 in selected solvents. In parenthesis the relative absorbance of the diketo conformers.

The *A*_DK_ values in the different solvents are reported in Table 2 next to the corresponding λ_DK_ (see values in parentheses). As previously observed for K2A23 (see reference [31] for a more exhaustive discussion), the non-monotonic dependence of both the λ_DK_ and the *A*_DK_ from the solvent polarity might reflect the complex equilibrium among structures of very different polarity, namely: the minimally polar *trans* diketo; the enols, endowed with intermediate polarity; and the maximally polar *cis* diketo conformer. The different ability of both the solvents and the K2T23 tautomers to form H-bonds further contributes to complicate the pattern.

#### Nuclear magnetic resonance spectroscopy

**Fig 3:**
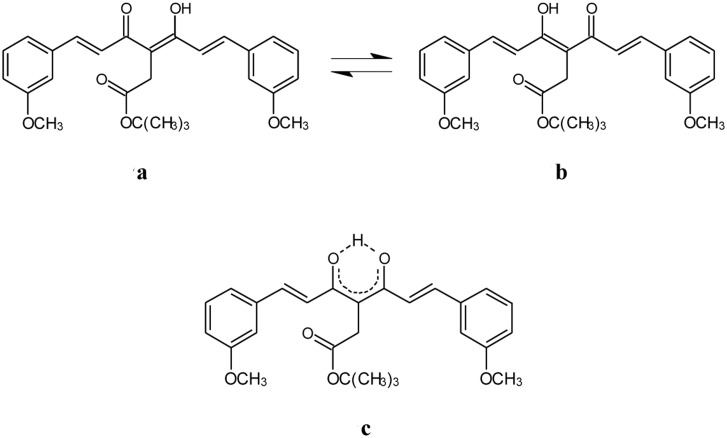
Tautomeric equilibrium between C_s_ and C_2v_ enol forms of K2T23. The asymmetric C_s_ structures a and b may interconvert through a C_2v_-like transition state, or the latter, symmetric structure c, may be more representative of the closed keto-enol conformer of K2T23 in the ground state.

Nuclear magnetic resonance spectroscopy was mainly devoted to investigate the keto-enol tautomerism in the ground state, as an independent confirmation of the results yielded by UV-Vis spectroscopy. For an exhaustive discussion on the ^1^H NMR spectra we remand to previous publications [28,35–37]. In curcuminoids, the prototropic equilibrium between the diketo and enol structures is slow on the NMR timescale, so that different NMR spectra can be obtained for the diketo and keto-enol tautomers participating in the equilibrium. Conversely, at least in the case of K2T23 and by means of ^1^H NMR measurements in solution we are neither able to discriminate among the different keto-enolic tautomers, nor among the diketo tautomers, due to the relatively faster proton kinetics characterizing these tautomerisms. More specifically, owing to K2T23 symmetry, the closed cis enol structures are intrinsically indistinguishable. Indeed, the two C_s_ structures depicted in Fig 3 (structures a and b) produce equal ^1^H NMR bands due to the identical phenyl rings. Moreover, we cannot extract any information about the nature of the keto-enol ring symmetry. Namely, two C_s_ symmetry keto-enols may be connected through a transition state with C_2v_ symmetry and separated by a very small barrier allowing fast proton tunneling between a and b, or the C_2v_ symmetry (see Fig 3, structure c) may be more representative of the K2T23 ground state.

**Fig 4:**
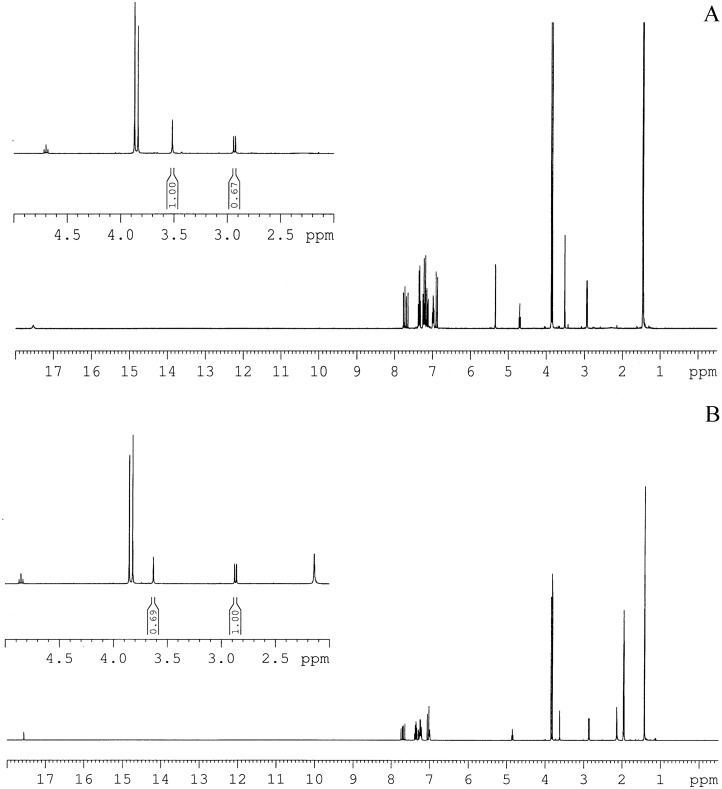
Exemplary NMR spectra of K2T23. ^1^H NMR spectra of K2T23 at room temperature in d_2_-Dichloromethane (upper panel) and d_3_-Acetonitrile (lower panel). In the insets the zoom of the region between 2 ppm and 5 ppm, where the peaks integrated to derive the fraction of compound in the diketo conformers, is presented.

According to the classically proposed vision, in simple β-diketones the closed keto-enols should be endowed with a C_s_ symmetry (i.e., the keto-enolic ring is asymmetric, with the enolic proton nearer to one than to the other oxygen) [38,39]. However, there has been a lively debate in the literature in the past forty years on whether this is a general rule, or rather depending on the specificity of the molecular species and environment the keto-enol ring may preferentially assume a C_2v_ symmetry (i.e., with the enol proton equidistant from the oxygens) [37–50]. A glance on the different theses proposed even for one of the simplest and most characterized β -diketones, acetyl-acetone, suggests that the question is non-trivial [38–42]. On the contrary, as mentioned above, we could clearly distinguish the diketo from the enol structures. Namely, as exemplarily shown in Fig 4 for the compound dissolved in dichloromethane and acetonitrile the value of *f*_DK_ could be determined as the ratio of the integrals of the doublet for CH_2_ in diketo form, falling at 2.87 ppm in dichloromethane and at 2.80 in acetonitrile, respectively, ^3^J = 7.0 Hz, versus the singlet for CH_2_ in keto-enol form, falling at 3.50 ppm in dichloromethane and 3.63 ppm in acetonitrile, respectively. The obtained *f*_DK_ are listed in Table 3. They roughly match the values of *A*_DK_ reported in Table 2, suggesting that assuming similar molar extinction coefficients at λ_DK_ and λ_KE_ is actually reasonable.

**Table 3:** NMR based assessment of K2T23 keto-enolic equilibrium.

Solvent	*f*_DK_
d_12_-cyclohexane	0.508
d_3_-chloroform	0.395
d_2_-Dichloromethane	0.396
d_3_-Acetonitrile	0.615
d_6_-Dimethylsulfoxide	0.481
d_4_-Methanol	0.470

Fraction of K2T23 in the diketo conformers in selected solvents as evaluated by NMR.

### Steady-state fluorescence

**Fig 5:**
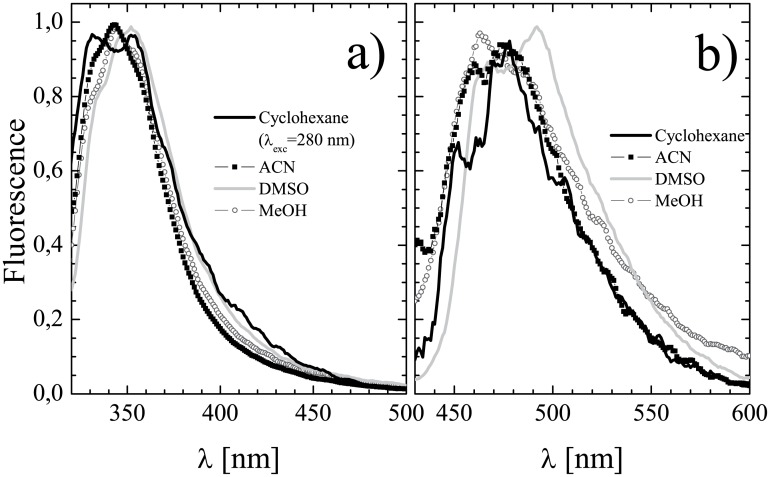
Fluorescence emission properties of K2T23. Emission spectra obtained upon excitation at a) the diketo, and b) the keto-enol, absorption peaks for K2T23 dissolved in cyclohexane (line), acetonitrile (squares), dimethylsulfoxide (crosses), and methanol (circles).

The steady-state fluorescence emission spectra were recorded for diluted (peak absorbance <0.1) solutions of K2T23 in the solvents of Table 1 both upon excitation at the peak wavelength of the diketo absorption band and at the peak of the keto-enol absorption band (see Table 2). In all the solvents, the emission band detected upon excitation at λ_DK_ is notably blue-shifted and much more intense with respect to the one detected upon excitation in the keto-enol absorption band. This result further supports the coexistence of two species in solution. The emission peak-wavelengths of the diketo and enol conformers in the various solvents are listed in Table 4. Exemplary emission spectra are shown in Fig 5. In general, the spectra are essentially structure-less and the spectral line-shape of both the diketo (Fig 5a) and the keto-enol (Fig 5b) conformers emission is poorly dependent on the environment. An exception is constituted by the compound dissolved in cyclohexane, in which two emission peaks at 330 nm and 353 nm are distinguishable upon excitation at 280 nm, i.e., at a wavelength intermediate between the two distinct absorption peaks resolved in this solvent within the diketo absorption band (see Table 2 and Fig 2). The above spectral features suggest that in this non-polar environment the absorption bands owning to the trans- and cis- diketo conformers are so sharp as to be spectrally resolvable (see [31], namely Fig 3 therein and the pertaining comment, for a discussion on the relationships between coexistence of different conformers of the same tautomer and intra-band structure). However, the barycenter of the emission band is localized around 345 nm, whereas in the other solvents for the diketo conformers the maximum emission peak shift is observed between methanol (λ_emi,DK_ = 343 nm) and chloroform (λ_emi,DK_ = 355 nm), and the full width at half maximum of the emission band remains between 46 nm (ethanol) and 58 nm (dimethylformamide). For the keto-enol conformers the maximum emission peak shift is observed between chloroform (λ_emi,KE_ = 469 nm) and dimethylsulfoxide (λ_emi,KE_ = 492 nm), and the full width at half maximum of the emission band remains between 65 nm (cyclohexane) and 81 nm (chloroform).

**Table 4:** Fluorescence emission maxima of K2T23.

Solvent	λ_emi,DK_ [nm] (Φ_Fl,DK_)	λ_emi,KE_ [nm] (Φ_Fl,KE_)
Cyclohexane	330, 353 (.243±.007)	476 (.0010±.0004)
Chloroform	355 (0.283±.005)	469 (0.0018±.0004)
Dichloromethane	345 (0.194±.004)	486 (0.0015±.0003)
Acetonitrile	343 (0.314±.003)	475 (0.0017±.0003)
Dimethylformamide	354 (0.157±.002)	487 (0.0033±.0005)
Dimethylsulfoxide	350 (0.125±.004)	492 (0.0035±.0004)
Isopropanol	343 (0.262±.003)	477 (0.0020±.0002)
Ethanol	344 (0.150±.005)	479 (0.0018±.0004)
Methanol	343 (0.152±.005)	474 (0.0022±.0004)

Emission peak wavelengths of the diketo (λ_emi,DK_, column 2) and keto-enol conformers (λ_emi,KE_, column 3) of K2T23 in the solvents of Table 1. The pertaining fluorescence quantum yields are reported in brackets.

The fluorescence quantum yields as estimated by comparison with dimethyl-popop are reported in brackets in Table 4. Those of the keto-enol conformers are generally very low and only slightly different from solvent to solvent. Actually they have values close to that measured for CURC in cyclohexane, where ESIPT is most efficient.

Moreover, the emission maxima are in all solvents but cyclohexane dramatically blue-shifted with respect to those recorded for CURC (the minimal shift is recorded in chloroform, and it amounts to as much as 34 nm). A similar phenomenology was observed in the case of dicinnamoylmethane (DCMeth), a CURC analogue lacking the methoxy and hydroxyl phenolic substituents, and attributed to the survival of the KEHB to solvent perturbation, thus to the presence of substantial amounts of closed cis-enol, in any environment [20]. Interestingly, both K2T23 [28] and DCMeth [46] exhibit crystal structures in which the enol proton is less localized on either of the two oxygen atoms of the β-diketo system with respect to CURC [44]. Actually, for DCMeth the crystal structure of the keto-enolic system approaches a C_2v_ configuration, in which the enolic proton is equidistant from the two oxygens. The reduced degree of symmetry observed for K2T23 in the crystal phase might be ascribed to the presence of the *tert*-butyl substituent. However, in solution the orientation of such moiety with respect to the keto-enolic ring is randomized, thus it is likely that the proton be more delocalized between the two oxygens in the solution phase. Moreover, the pKa of the enol proton is estimated to be ≈9 for K2T23 [28] and ≈7.5 for CURC [29]. This much tighter bond of the enolic proton is shared by DCMeth [20]. In conclusion, a blue shifted and less solvent-dependent emission spectrum, combined with a low quantum yield, seems to correlate with a tighter KEHB, thus with the presence of substantial amounts of closed cis-enol, in any environment. In turn, at least in curcuminoids, the compounds showing tighter KEHB share more symmetric keto-enol system configurations (approaching the C_2v_ structure). Fluorescence spectroscopy, possibly combined to high-resolution measurements of the time-resolved excited state dynamics, might thus be exploited to shed new light on the debated question of keto-enol ring symmetry in β-diketones.

The diketo conformers are endowed in any environment with fluorescence quantum yields higher than those exhibited by either CURC or any other curcuminoid we previously characterized. This confirms that the diketo *S*_1_ excited state is much more stable towards non-radiative decay than that of the keto-enol conformers.

**Fig 6:**
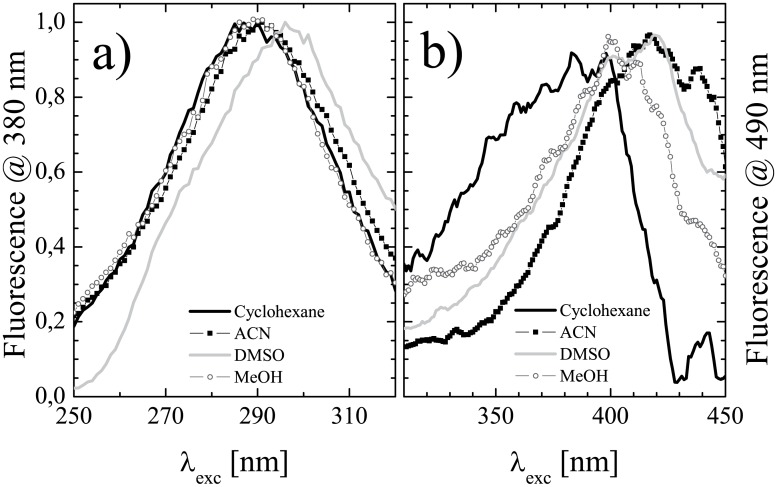
Fluorescence excitation spectra of K2T23. Excitation spectra of K2T23 dissolved in cyclohexane (line), acetonitrile (squares), dimethylsulfoxide (crosses), and methanol (circles), obtained by monitoring the fluorescence intensity: a) at λ_obs,DK_ = 380 nm; b) at λ_obs,KE_ = 490 nm.

For the same samples as above, the excitation spectra were also measured, by monitoring the fluorescence emission intensity at λ_obs,DK_ = 380 nm and at λ_obs,KE_ = 490 nm. The corresponding peak excitation wavelengths are reported in Table 5, columns 2 and 3, respectively. Exemplary excitation spectra are displayed in Fig 6. The excitation spectra obtained at λ_obs,DK_ displayed maxima around λ_DK_, while those obtained at at λ_obs,KE_ were peaked around λ_KE_. This observation further supports the assignation of the two absorption bands to different conformers of K2T23, exhibiting independent decay mechanisms. However, it is worth noting that the excitation spectra do not exactly superimpose to the corresponding absorption spectra, which supports the co-existence of different tautomers of both the diketo and the keto-enol structure, endowed with slightly different quantum yields and/or emission maxima. To this regard, the splitting of the rather unstructured diketo absorption band recorded in chloroform into two well separated excitation bands is emblematic.

**Table 5:** Fluorescence excitation maxima of K2T23.

Solvent	λ_exc,DK_ [nm]	λ_exc,KE_ [nm]
Cyclohexane	290	388
Chloroform	269, 315	381
Dichloromethane	297	419
Acetonitrile	291	417
Dimethylformamide	294	413
Dimethylsulfoxide	296	420
Isopropanol	290	410
Ethanol	290	405
Methanol	290	404

Excitation peak wavelengths of the diketo (λ_exc,DK_, column 2) and keto-enol conformers (λ_exc,KE_, column 3) of K2T23 in the solvents of Table 1.

### Excited-state dynamics

**Fig 7:**
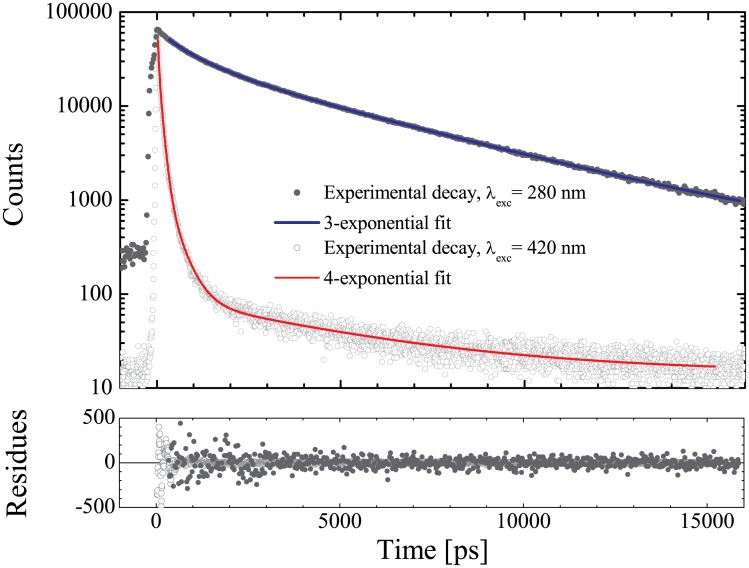
Fluorescence decay patterns of K2T23. Exemplary decay distributions obtained for K2T23 dissolved in isopropanol upon excitation at 280 nm (full dots), and 420 nm (empty circles). The continuous blue line represents the best-fitting curve for the former according to a tree-exponential decay function, while the red line is the best-fitting curve for the latter to a four-exponential decay model. The corresponding residual plots are reported in the lower panel.

The fluorescence decay distributions of K2T23 were reconstructed upon excitation at 280 nm (in proximity of the absorption peak of the diketo conformers) and at 420 nm (in proximity of the absorption peak of the keto-enol conformers) in all the solvents of Table 1. The decay fitting parameters obtained in the two instances are summarized in Tables 6 and 7, respectively. Exemplary decays, namely those obtained for the compound dissolved in isopropanol, a solvent in which all the decay transients have been resolved, are plotted in Fig 7 for excitation at both 280 nm and 420 nm. We first consider the decays obtained with excitation at 420 nm, which can be interpreted on the base of our previous experience on the excited-state dynamics exhibited by non-carbonyl-substituted curcuminoids. According to Table 7, the decay patterns result to be essentially double-exponential in all the solvents except dimethylsulfoxide. More than 60% of the compound decays with a lifetime τ_1_ shorter than ≈50 ps (the value detected in dimethylformamide), and comparable with the TCSPC system temporal resolution (30 ps FWHM). It is worth noting that transients of lifetime comparable (i.e. slightly longer or slightly shorter than) the instrumental response function (IRF) variance, σ^2^, are detected with lifetime somewhat larger than the real one. The latter can be only estimated by means of the equation $\tau =\sqrt{{\left(\tau_{measured}\right)}^{2}-\sigma^{2}}$. Moreover, the corresponding relative amplitude are underestimated. However, if a decay transient is definitely shorter than the IRF, it is integrated within the distribution and becomes undetectable. Thus, the actual value of τ_1_ cannot be much shorter than 30 ps. For the above reasons, although we chose to report in Table 7 the values of τ_1_ yielded by the fit in order to provide the reader with complete information on the minimization algorithm stability, one should keep in mind that these values are barely indicative. Consequently, we put the values yielded by the fits in brackets, and added the notation ≤30 ps.

**Table 6:** Fluorescence decay of the K2T23 diketo conformers.

Solvent	τ_1_ (*f*_1_)	τ_2_ (*f*_2_)	τ_3_ (*f*_3_)
Cyclohexane	259 ± 29 (.26)	2312 ± 42 (.74)	--
Chloroform	252 ± 20 (.46)	1866 ± 128 (.35)	3540 67 (.19)
Dichloromethane	283 ± 27 (.36)	1723 ± 108 (.36)	4090 ± 94 (.28)
Acetonitrile	229 ± 33 (.14)	1478 ± 91 (.37)	4255 ± 249 (.49)
Dimethylformamide	154 ± 36 (.19)	1570 ± 117 (.32)	4314 ± 189 (.49)
Dimethylsulfoxide	567 ± 11 (.38)	--	4076 ± 70 (.62)
Isopropanol	422 ± 42 (.20)	1268 ± 106 (.33)	4409 ± 214 (.47)
Ethanol	245 ± 34 (.22)	1724 ± 84 (.33)	4104 ± 124 (.45)
Methanol	212 ± 23 (.09)	1748 ± 95 (.31)	4025 ± 146 (.60)

Decay times and relative amplitudes of K2T23 in the Table 1 solvents upon excitation at 280 nm.

**Table 7:** Fluorescence decay of the K2T23 enol conformers.

Solvent	τ_1_ (*f*_1_)	τ_2_ (*f*_2_)	τ_3_ (*f*_3_)	τ_4_ (*f*_4_)
Cyclohexane	≤30 ps (19 ± 2) (.72)	99 ± 2 (.24)	535 ± 12 (.02)	3005 ± 12 (.01)
Chloroform	≤30 ps (21 ± 1) (.75)	103 ± 1 (.24)	480 ± 13 (.01)	4112 ± 90 (<.01)
Dichloromethane	≤30 ps (26 ± 1) (.77)	111 ± 2 (.22)	462 ± 13 (.01)	
Acetonitrile	≤30 ps (29 ± 1) (.78)	116 ± 2 (.21)	501 ± 15 (.01)	
Dimethylformamide	51 ± 3 (.74)	125 ± 14 (.25)	428 ± 64 (.01)	4520 ± 515 (<.01)
Dimethylsulfoxide		94 ± 1 (.97)	461 ± 24 (.03)	4281 ± 180 (<.01)
Isopropanol	≤30 ps (28 ± 2) (.60)	91 ± 3 (.38)	336 ± 8 (.02)	4197 ± 177 (<.01)
Ethanol	≤30 ps (34 ± 1) (.67)	100 ± 2 (.31)	355 ± 9 (.02)	
Methanol	≤30 ps (29 ± 1) (.68)	95 ± 1 (.30)	322 ± 8 (.02)	

Decay times and relative amplitudes of K2T23 in the Table 1 solvents upon excitation at 420 nm.

However, besides the above technicalities, the existence of a transient of the order of few tens of picoseconds and relative amplitude >60% in all the decays is unquestionable as can be easily appreciated by Fig 7. The second component of the decays obtained by excitation of the samples at 420 nm has time constant τ_2_ ≈ 100 ps. Besides these dominating short-lived decays, a third transient with lifetime 322 ps ≤ τ_3_ ≤ 535 ps is resolved in all the solvents, although with relative amplitudes ≤ 0.03. In cyclohexane, chloroform, dimethylformamide, dimethylsulfoxide, and isopropanol a further long-lived (> 3ns) component is also resolved, whose relative amplitude is nevertheless negligible (≤ 0.01). Once again, the above features are similar to those observed for DCMeth and detailed in a previous article of ours [20]. We are tempted to ascribe this similarity to the fact that for K2T23 the configuration of the keto-enol system in solution approaches the C_2v_ symmetry distinctive of DCMeth even in the crystal phase, by contrast with CURC and most other curcuminoids, including some we occurred to examine in the past, which shared very different excited-state dynamics [44–50]. Accordingly, we proceed to attribute an *S*_1_-state deactivation pathway to each of the decay components detected for K2T23 by analogy to DCMeth, remanding to the above-quoted reference for a full discussion on the decay photophysics of the two compounds. Namely, we interpret the τ_1_ and τ_2_ components as the fingerprints of two different ESIPT mechanisms, i.e., the one occurring through the direct transfer pathway and the one occurring through the reaction coordinate pathway, respectively. We remind that the occurrence of direct (i.e., non-solvent rearrangement moderated) ESIPT in any environment is a consequence of the presence of substantial amounts of closed *cis-* enol conformer, thus ultimately of an exceptional stability of the KEHB towards solvent perturbations. Conversely, in CURC, as well as in the other non-carbonyl substituted compounds tested in the past, the closed *cis-* enol conformation was assumed, and direct ESIPT took place, only in a rather inert solvent such as cyclohexane. By consequence, the fraction of K2T23 in the enol conformation exhibits much faster *S*_1_-state deactivation dynamics with respect to CURC. For the sake of completeness, we finally exploit the comparison of the K2T23 decays with those of DCMeth and K2A23 to identify the decay mechanisms corresponding to τ_3_ and τ_4_ with reketonization and with the emission of out-of-peak-excited diketo conformers, respectively [20,31].

Upon excitation at 280 nm, the excited-state dynamics of the diketo conformers should be primarily unraveled. The short-time behavior of such conformers is dominated by a decay transient whose time constant is similar to the τ_3_ component detected for the enol structures of K2T23, as well as for both the enol and diketo conformers of the closely related curcuminoid K2A23 [31]. As originally proposed for the latter compound [31], we ascribe this transient to excited-state tautomerization from the diketo to the enol structure, which should occur with similar rate and probability as reketonization due to the high abundance of K2T23 in diketo conformers in the ground state. The long-time behavior of the diketo decay distributions is very similar to that exhibited by the closely correlated compound K2A23 when dissolved in both acetonitrile and methanol and excited at 280 nm. Two different components τ_2_ ≈ 2 ns and τ_3_ ≈ 4 ns are resolved in all the solvents except the minimally polar cyclohexane, where only τ_2_ is detected, and the maximally polar dimethylsulfoxide, in which only τ_3_ survives. The sum of the relative amplitudes of the two components represents >50% of the entire decay. This corroborates our hypothesis that both components are emitted by diketo conformers [20,21,31]. Moreover, in such studies we attributed, somewhat arbitrarily, the ≈ 2 ns component to the emission of the *trans*- diketo conformer and the ≈ 4 ns to the decay of the *cis*- diketo conformer, on the basis of the fact that the former was preferentially observed in relatively non-polar solvents, whereas the latter was typically detected in polar solvents. Because the relative amplitude of τ_3_ monotonically increases with the solvent polarity, the present results confirm the above speculations. On the other hand, the fact that in most environments both τ_2_ and τ_3_ are detected suggests that, similarly to what observed for K2A23, also for K2T23 the difference in polarity between the *trans*- and *cis*- diketo conformer is reduced in comparison to that proper of curcuminoids lacking a carbonyl substituent. If for the former compound this reduction was proposed to be related to the insertion of a notably polar moiety such as the carboxylic acid, which might induce an increase in polarity of the *trans-* diketo conformer, in the case of K2T23 a similar role might be played by the rather non-polar *tert*-butyl substituent, which should decrease the overall polarity of the *cis-* diketo conformer.

Finally, we note that the very fast decay transient observed for both the diketo and the enol conformers of K2A23 and attributed to an intermolecular proton transfer mechanism involving the acidic proton of the carboxyl moiety [31] is not detected for K2T23 upon excitation in correspondence of the diketo conformers absorption peak. This evidence supports the attribution of such a decay component to a mechanism directly involving the carboxylic acid substituent, rather than to a general consequence of carbonyl substitutions.

### Spectrofluorimetric detection of photosensitized ROS generation

As a preliminary attempt to assess the effects of the shifted of keto-enolic equilibrium towards the diketo conformers in K2T23 with respect to CURC on its photosensitizing potency, we tested the ability of the compound to generate singlet oxygen (^1^O_2_) through the canonical type II photosensitizing reaction. To this aim, we applied to both curcuminoids the well-established assay based on selective fluorescence quenching of the dye 9,10-dimethylanthracene by this ROS. Before reporting on the outcomes of this assay, it is worth mentioning that the mechanisms of action of curcuminoids as photosensitized cytotoxic agents are still under investigation and the very modest ^1^O_2_ production of curcumin itself can hardly account for its notable potency against both bacterial and cancer cells. Other ROS might be produced in more relevant amounts, and their production might be notably increased by shifting the keto-enolic equilibrium towards the diketo conformers, without any effect on the 9,10-dimethylanthracene fluorescence. Indeed, it should be considered that the oxidation of 9,10-dimethylanthracene leading to its conversion into the non-fluorescent dye 9,10-endoperoxide occurs with negligibly lower efficiency if ROS different other than ^1^O_2_ are used as the oxidizing species [51]. Alternatively, as suggested by other authors, the photosensitizing activity of CURC might be mediated by an excited-state oxidation reaction of the photosensitizer itself, with formation of a long-lifetime CURC radical [52,53]. In the latter case, chemical and structural details of a curcuminoid would be of major importance as well as a long excited state lifetime in order to establish its photosensitizing efficacy. Thus, the data presented below are far from constituting an exhaustive characterization of the relative photo-toxicity of K2T23 with respect to CURC.

In Table 8 we report the fractional fluorescence decrease of 9,10-dimethylanthracene upon illumination of the samples added with 0.5 μM CURC with light at 280 nm and 420 nm for 10 min. In all the tested solvents, we measure substantial fluorescence decreases. Interestingly, the UV light appears more effective in inducing the ROS generation, even though the HOMO-LUMO transition for CURC occurs within the 420 nm-peaked band. Thus, either excitation of the compound on a higher electronic energy level might lead to more efficient intersystem crossing or ROS generation might be triggered by photochemical reactions different from the conventional singlet-triplet interconversion mediated by molecular oxygen exploited by most photosensitizers, including porphyrins, anthracyclines and chlorines.

**Table 8:** Spectrofluorimetric assessment of singlet oxygen generation by CURC and K2T23.

Solvent	[F(0)-F(10 min @ 280 nm)]/F(0)	[F(0)-F(10 min @ 420 nm)]/F(0)
Acetonitrile	0.83	0.78 (0.04)
Dimethylformamide	0.55	0.59 (0.08)
Methanol	0.39	0.33 (0.03)
Ethanol	0.44	0.35 (0.02)

Fractional fluorescence decrease of 9,10-dimethylanthracene in 10:1 dimethylanthracene:CURC mixtures in the solvents indicated in column 1 upon illumination of the sample with light at 280 nm (column 2) and 420 nm (column 3) for 10 minutes (see text for details). The results in parenthesis in column 3 were obtained with K2T23 as the photosensitizer.

We remind that the fluorescence of solutions of pure 9,10-dimethylanthracene (i.e., in the absence of photosensitizer) did not change after exposure to the same light doses. Accordingly, we interpret the fluorescence decrease as an evidence that substantial amounts of ^1^O_2_ are produced. However when we performed the same measurements using 0.5 μM K2T23 as the photosensitizer, we did not detect any appreciable decrease in the 9,10-dimethylanthracene fluorescence upon illumination with light at 280 nm, and a barely detectable, although systematic, decrease upon illumination at 420 nm (the values of fractional fluorescence decrease are in parenthesis in Table 8, column 3, but they are not quantitatively reliable due to the small difference between the spectra recorded before and after illumination). If the latter result was expectable due to the much shorter average excited-state lifetime of K2T23 keto-enolic conformers compared to CURC, the former calls for further investigations. Although other authors have proposed that in CURC the generation of ROS is promoted by formation of a CURC radical upon deprotonation of the phenolic hydroxyl substituents [52], the lack of the phenolic hydroxyl groups cannot be invoked as the cause of K2T23 inability to photosensitize ^1^O_2_ production. Indeed, the ^1^O_2_ production efficiency of bis-dehydroxy-curcumin and dimethoxy-curcumin were measured by direct detection of the ^1^O_2_ luminescence at 1270 nm and resulted comparable and even higher than that of the parent compound in both acetonitrile and methanol [22]. Deprotonation and formation of curcuminoid radicals might rather occur at the enol proton, which was actually identified as the most acidic for CURC by some authors [29]. Indeed, the results obtained herein seems to indicate that the enol structure is needed in order for both CURC and K2T23 to generate ^1^O_2_.

## Conclusions

In the present work the tautomeric equilibrium between the keto-enolic and diketo conformers of the synthetic curcuminoid K2T23, functionalized with a *tert*-butyl ester moiety at the carbonyl, was assessed in several organic solvents by UV-visible absorption and by nuclear magnetic resonance. In analogy with a previously characterized curcuminoid substituted at the same position with a carboxylic acid moiety, the tautomeric equilibrium of K2T23 shifted toward the diketo conformers with respect to natural curcumin and other curcuminoids lacking substitution at the carbonyl ring. Steady state fluorescence spectroscopy and time-correlated single-photon counting measurements showed that the fluorescent emission of the diketo fraction of K2T23 is more intense and notably longer-lived with respect to that emitted by any of the several non-carbonyl substituted curcuminoids that we had previously examined, including CURC itself, as well as by the fraction of K2T23 in the keto-enol conformers. This finding confirms that operating substitutions at the carbonyl allows shifting the equilibrium towards the diketo structures, thereby stabilizing their *S*_1_-excited state. Although preliminary measurements undertaken to evaluate the ability of K2T23 to photosensitize the production of singlet oxygen led to disappointing results, further investigations are needed to elucidate the molecular mechanisms at the basis of CURC phototoxicity and to conclude that acting on the keto-enolic equilibrium is not an efficient strategy to maximize the photosensitizing potential of this class of model drug compounds.
